# Advances in asymmetric organocatalysis over the last 10 years

**DOI:** 10.1038/s41467-020-17580-z

**Published:** 2020-07-29

**Authors:** Shao-Hua Xiang, Bin Tan

**Affiliations:** 1 Shenzhen Grubbs Institute and Department of Chemistry, Southern University of Science and Technology, Shenzhen, 518055 China; 2 Academy for Advanced Interdisciplinary Studies, Southern University of Science and Technology, Shenzhen, 518055 China

**Keywords:** Asymmetric catalysis, Catalytic mechanisms, Organocatalysis

## Abstract

Beyond esoteric interest, organocatalysis has now become one major pillar of asymmetric catalysis. Here, we discuss how new activation modes are conquering challenging stereoselective transformations and the recent integration of organocatalysis with emerging photo- and electrocatalysis, as well as artificial intelligence.

## Abstract (web summary)

Organocatalysis has become a major pillar of (asymmetric) catalysis. Here, the authors discuss recent trends in organocatalytic activation modes for challenging stereoselective transformations and the emerging integration with other fields, such as photoredox catalysis and electrosynthesis.

Impressed by the catalytic systems occurring in nature, chemists looked into imitating biocatalytic processes. This led to the birth of organocatalysis in the late 1990s^1,2^ where chiral organic molecules bear the minimal functionalities to mimic biocatalysts and effect asymmetric reactions. Today, organocatalysis is one of the most thriving research domains in contemporary organic synthesis and covers a series of classic catalytic modes^3–10^ alongside numerous other valuable yet challenging chemical transformations. Although the green feature of organocatalysis is frequently disputed owing to difficulties with catalyst recycling and/or high catalyst loading, this field has evolved to parallel and complement transition-metal catalysis in assembling chiral molecules^11^. In the last decade, ~1500 publications on organocatalysis have been published each year. Prominently, the efforts to unify organocatalysis and photoredox catalysis by MacMillan in 2008 symbolize another milestone in enantioselective functionalization of otherwise difficult molecules^12^. These landmark discoveries refashion retrosynthesis and are revolutionizing other chemistry domains such as natural product synthesis, drug discovery, and fine chemicals production. Accordingly, enantioselective organocatalysis is elected as one of the 10 emerging technologies in Chemistry with potential to make our planet more sustainable by IUPAC on its 100th anniversary of foundation in 2019^13^.

## Development of new catalytic strategies

**Fig. 1:**
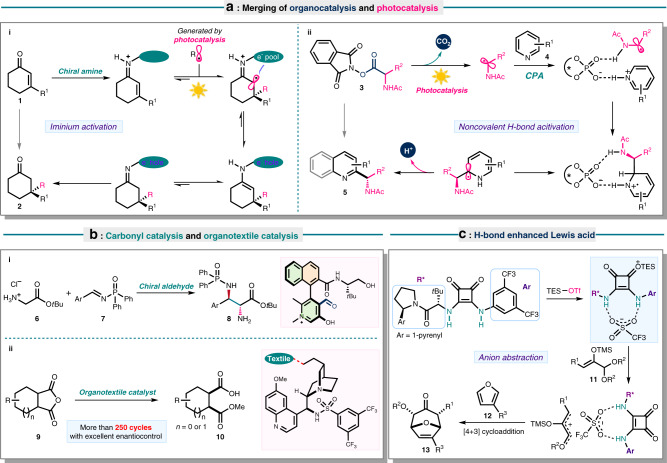
Development of new catalytic strategies. **a** Combination of organocatalysis with photocatalysis. **b** Carbonyl catalysis and organotextile catalysis. **c** Lewis acid enhancement by H-bond donor catalysis.

Despite the potentially green features of photocatalysis, practitioners in this area are often challenged by the achievement of full stereochemical control in a photochemical reaction due to the inherently high reactivity of the radicals. This scenario was overturned in 2008 after MacMillan and co-workers reported the inventive merging of enamine-mediated covalent catalysis and photoredox catalysis. In 2016, Melchiorre^14,15^ applied iminium activation in photocatalysis to deliver *β*,*β*-disubstituted cyclic enones **2** with very high enantiopurity in the asymmetric construction of quaternary carbon stereocenters (Fig. 1a, reaction i)^16^. The dual catalytic system with non-covalent organocatalysis was successfully implemented in 2013 by Knowles’ group^17^, who pioneered the use of chiral phosphoric acid (CPA)^5,6,18^ in asymmetric photocatalysis^19^. Harnessing this verified catalytic mode, Phipps’ group accomplished an intermolecular decarboxylative cross-coupling of *N*-acetyl-*L*-phenylalanine **3** and 4-methylquinoline **4** (Fig. 1a, reaction ii)^20^. Thereafter, a novel photosensitizer dicyanopyrazine-derived chromophore (DPZ) was developed by Jiang’s group as a substitute of expensive iridium photocatalysts^21^.

Activating carbonyl group with chiral secondary amine underpins the working principle of enamine catalysis for enantioselective functionalization of aldehydes or ketones, whereas carbonyl catalysis represents the reversal of this strategy. By analogy to pyridoxal-dependent enzymes^22^ in carbonyl catalysis, Zhao’s group innovatively disclosed a class of organic small molecule-carbonyl catalysts in 2018 (Fig. 1b, reaction i)^23^. This well-defined strategy enabled asymmetric Mannich reaction of glycinate **6** with *N*-diphenylphosphinyl imines **7** in the presence of an N-quaternized pyridoxal catalyst, furnishing synthetically useful *a*,*β*-diamino acid esters **8** in high efficiency with remarkable stereocontrol. Since then, substantial research efforts culminated in a wide array of asymmetric transformations by exploiting chiral aldehydes as the reliable amine activation catalysts^24^.

List group introduced organotextile catalysis built upon photochemical fixation of organocatalysts on textile nylon via a radical course (Fig. 1b, reaction ii)^25^. The organocatalysts exhibited excellent compatibility with polyamide to provide heterogeneous organocatalysts harbouring Lewis bases, Brønsted acids and chiral cinchona alkaloid derivatives. Excellent stability and efficiency comparable to the corresponding homogeneous catalysts were evidenced. This approach addressed the recyclability issue in classic organocatalysis with near-perfect enantioselectivity preserved after >250 asymmetric catalytic cycles. This impressive robustness of organotextile catalysts portends successful applications in industrial production.

Asymmetric organocatalytic nucleophilic additions are usually undertaken by hydrogen-bond catalysis. Given the modest acidity of chiral H-donors such as thioureas, squaramides, and guanidinium ions, the incompatibility of some substrate classes persists, which could be rectified by introduction of a Lewis acid as co-catalyst. Inspired by the reactivity enhancement through interaction with more weakly coordinating ligands, an extremely active Lewis acid catalyst generated by in situ silylation from chiral C-H acids was designed by List and coworkers to promote asymmetric Diels-Alder reaction^26^. One year later, a H-bond catalyst-assisted Lewis acid enhancement strategy was ingeniously utilized by Jacobson’s group with squaramide as a weak H-donor (Fig. 1c)^27^. Control experiments demonstrated the high reactivity of silyl triflate–squaramide ion pair and NMR analysis shed light on the interaction between TESOTf (triethylsilyl trifluoromethanesulfonate) and squaramide wherein the latter establishes an adequate spatial environment for stereoinduction. These chiral active species readily mediate processes with heteroatom-stabilized cations to deliver Mukaiyama-type aldol reaction and [4 + 3] cycloaddition of enolates **11** with furan derivatives **12** in a highly stereoselective manner.

## Target-oriented developments in organocatalysis

**Fig. 2:**
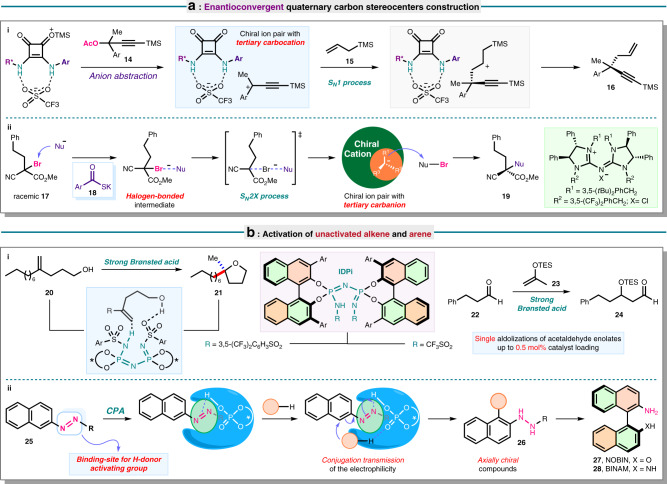
Important target-oriented developments. **a** Chiral ion pair enabled quaternary carbon stereocenters construction in enantioconvergent manner. **b** Application in inactive alkene and arene activation.

The catalytic enantioselective formation of fully substituted carbon stereocenters remains a challenging synthetic pursuit. Contrary to a S_N_2-type nucleophilic substitution reaction, enantio-differentiating addition in S_N_1-type pathway is challenged by the high reactivity of the prochiral carbocationic intermediates, as it appears evident in organocatalytic nucleophilic substitution of racemic tertiary electrophiles in an enantioconvergent fashion. To this end, the capability of the silyl triflate–squaramide ion pair to abstract an acetoxy anion^27^ was favorably leveraged in a catalytic enantioselective S_N_1 reaction by Jacobson’s group using racemic substrates with an acetoxy leaving group (Fig. 2a, reaction i)^28^. This work highlighted a stabilization of carbocation intermediate by non-covalent interaction with the chiral squaramide-embedded anion instead of a specific heteroatom interaction. While engendering effective enantio-discrimination for nucleophilic attack, the chiral ion pair also circumvents undesired elimination and rearrangement by-products. Tan’s group made a further stride in 2019 employing a chiral cationic pentanidium catalyst, which involves a different catalytic process (Fig. 2a, reaction ii)^29^. While both methods share a similar key formation of stabilized chiral ion pair, in Tan’s work a halogenophilic S_N_2X pathway with halogen leaving group is operative. The encumbered carbon center directs nucleophilic attack to electrophilic halogen atom, aided by halogen-bonding effect provided by two germinal electron-withdrawing groups.

Beyond imine and carbonyl substrates, the weak basicity and lack of binding site of unbiased alkenes often counterpoise the potency of chiral Brønsted acid catalysis. Consequently, a highly acidic and structurally well-defined chiral organic acid, which can functionalize this class of poorly active molecules is enthralling. List’s group devised a chiral imidodiphosphorimidate (IDPi) catalyst, which accomplished the straightforward enantioselective hydroalkoxylation of sterically and electronically unbiased alkenol **20**^30^. Density functional theory calculations supported a mechanism involving initiation of the reaction by alkene protonation, whereas the rigid and narrow chiral cavity is beneficial for enantiocontrolled cyclization. Catalytic single aldolizations of acetaldehyde enolates **23** were realized asymmetrically shortly after by virtue of IDPi catalyst without competing oligomerization (Fig. 2b, reaction i)^31^. Remarkable enantiopurity of β-hydroxy aldehyde **24** building blocks was maintained at 0.5 mol% catalyst loading. In the context of the more exacting arene functionalization, our group overrode the high dearomatization barrier through judicious installation of an azo group. The interaction of azo with CPAs via H-bond boosted the system electrophilicity and allowed arene activation via conjugation transmission (Fig. 2b, reaction ii)^32^.

Intensive research efforts further expanded the boundary of organocatalysis to generate atropisomers. Our group has been devoted toward asymmetric construction of axially chiral frameworks including the privileged NOBIN **27** and BINAM **28** by means of organocatalysis^33^, whereas Miller et al.^34^ engineered short peptides as minimalistic bio-mimetic catalysts involving H-bond catalysis. The exploitation of organocatalysts and ligands featuring an axially chiral element in turn accelerates the advance of organocatalysis, bringing in efficient solutions to longstanding challenges. These include multicomponent reactions (MCRs)^35^ such as four-component Ugi reaction and Passerini reaction known for high step and atom economy, the much valued attributes in diversity-oriented synthesis of complex molecules. The asymmetric variants have long remained unknown owing to the complexity of the mechanism as well as strong background and competing reactions^36^. Recently, enantiocontrol of the classical Ugi reaction was tackled with CPAs^37,38^. The H-bond interaction between CPA and acid component not only enhances the acidity of CPA and inhibits background reaction, but also strengthens the nucleophilicity of acid for attack of imine.

## Outlook

This 10 years’ advent of organocatalysis witnessed huge achievements in resolving enduring synthetic challenges as well as discovery of new catalytic modes, reaction types and advantageous integration with disparate reaction types. A sustained growth of this field is anticipated with refinement along several directions. First, most organocatalysts remain substandard compared with metal catalysts particularly for substrates without binding point. In this regard, stronger chiral Brønsted acids or Lewis acid enhancement tactics could enhance the potential of typical H-bond catalysis. More promisingly, asymmetric halogen, chalcogen, and pnictogen bonding catalysis may provide a new avenue in activation of inert substrates. Alternatively, merging organocatalysis with other synthetic strategies could develop into novel synthetic approaches. Parallel to the tremendous success in photocatalysis, the potential of electrocatalysis as another source of radical partners is likely to be implemented in organocatalytic reactions, however, the practical realization still remains in its infancy. Moreover, translating organocatalysis into industrial settings require efforts to improve both catalyst efficiency (in order to decrease the catalyst loading) and recyclability. Last but not least, introduction of machine learning into organocatalysis is underway. Although Denmark’s group has realized an accurate prediction of the selectivity of chiral phosphoric acid catalysts, reported models were confined to thio- and *N*-acylimine substrates^39^. There remains a long way before machine learning will be established as handy daily tool for chemists working in this field.
